# Surgical Excision Versus Medical Management of Primary Breast Lymphoma: A Case Report

**DOI:** 10.7759/cureus.32802

**Published:** 2022-12-21

**Authors:** Fadi Alyass, Laura A Ray

**Affiliations:** 1 Internal Medicine, Piedmont Macon, Macon, USA; 2 Graduate Medical Education, Mercer University School of Medicine, Macon, USA; 3 Breast Surgery, General Surgery, Piedmont Macon, Macon, USA

**Keywords:** rare breast mass, diffuse large b-cell lymphoma (dlbcl), general surgery and breast cancer, screening mammogram, extranodal low grade b-cell lymphoma

## Abstract

Lymphoma of the breast accounts for 0.4-0.5% of all breast-located cancers and is found in a similar fashion to breast cancers. Here we present a 74-year-old woman who presented for a biopsy of a breast mass found on a routine mammogram, which was found to be a primary breast lymphoma. According to current practice guidelines, medical therapy is favorable for the definitive management of primary breast lymphomas. However, biopsy specimen cytology found neoplastic cells positive for human germinal center-associated lymphoma, a nonspecific marker for various types of lymphomas. Without a definitive classification of lymphoma, optimal medical therapy could not be achieved. Therefore, a decision was made to undergo a lumpectomy of the mass, which yielded a specimen that was found pathologically favorable for diffuse large B-cell lymphoma. With this information, the patient was referred to follow-up oncology for adjuvant chemotherapy and radiotherapy.

## Introduction

Uncommonly, a breast mass may be found to be a primary breast lymphoma (PBL) with lymphatic spread to neighboring lymph nodes or axillary lymph nodes mimicking primary breast carcinoma prior to histopathological or cytological investigation [1]. Establishing a diagnosis of both primary breast carcinoma and PBL is similar [2,3]. Both pathologies are found with screening mammography, scored according to Breast Imaging Reporting and Data System (BIRADS), followed by breast ultrasound and biopsy. Standard of care for a non-Hodgkin’s lymphoma type PBL involves various chemotherapeutical options in the neoadjuvant or adjuvant periods following surgical excision of the mass. We present a case of low-grade follicular lymphoma of the breast with breast and axillary lymphadenopathy treated with breast-conserving partial mastectomy, adjuvant chemotherapy, and radiotherapy.

## Case presentation

A 74-year-old woman with a past medical history of coronary artery disease, hyperlipidemia, hypertension, and peripheral neuropathy presented for the management of a breast mass. The patient previously had been found to be developing left breast focal asymmetry on a screening mammogram in June 2022. Nine days later, the patient underwent a diagnostic ultrasound of the breast with diagnostic mammography, which revealed a focal distortion in the left lower breast at the 4 o’clock position, 3 cm from the nipple, that was described as a heterogenous hypoechoic mass-like lesion measuring 13 × 7 × 20 mm.

**Figure 1:**
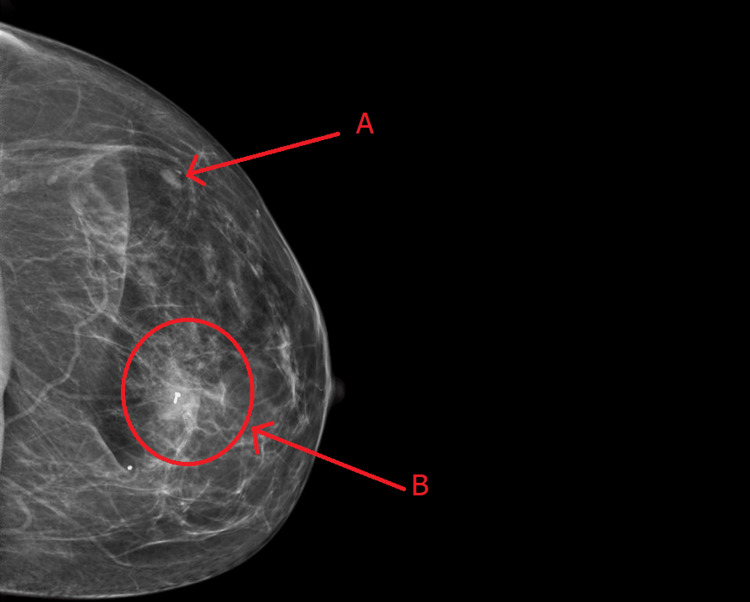
Diagnostic mammography tomosynthesis of the left breast. Label A: arrow denotes the mass at the 2 o’clock position, an enlarged intramammary lymph node. Label B: circle following the arrow denotes mass at the 4 o’clock position, a heterogenous, hypoechoic mass-like lesion. A bright spot seen in the mass indicates a biopsy clip.

The radiographic findings also identified an oval-shaped nodular density in the left outer breast, which was found to be an enlarged intramammary lymph node as well as an enlarged axillary lymph node. The patient’s findings were consistent with a score of BIRADS 4, which warranted a tissue biopsy and diagnosis for the breast mass. The patient subsequently underwent a left breast biopsy with the placement of biopsy clips. Figure 1 shows a mass-like lesion, an intramammary lymph node, and biopsy clips.

Left breast core biopsy showed B-cell colonization supporting a low-grade B-cell lymphoma with suspicion for a marginal zone lymphoma with follicular colonization or follicular lymphoma. Ancillary studies using fluorescence in situ hybridization (FISH) showed human germinal center-associated lymphoma (HGAL) being positive in a minority of cells between follicles with negative findings for B-cell leukemia/lymphoma protein (BCL) and B-cell leukemia/lymphoma protein 2 (BCL2) rearrangements, which are frequently found in follicular lymphomas. HGAL is considered an early marker for follicular lymphomas, Burkitt lymphomas, mediastinal large B lymphomas, and diffuse large B-cell lymphomas (DLBCLs) [4-6]. There was no evidence for MALT1 rearrangement, which is found in marginal-zone lymphomas, and the pathological findings did not show evidence for classic Hodgkin’s lymphoma.

Traditionally, surgical excision is not a widely used facet of care in non-Hodgkin’s lymphomas due to the efficacy of medical treatment [2,3,7]. While it is preferable to obtain adequate biopsy material so that lymphoma classification can be established, this was not the case for the patient. The findings on the core biopsy sample were ultimately indeterminate for classification; therefore, surgical excision was planned. This option allowed additional surgical cytology datapoints of the excised mass to help guide treatment, which was lacking in the initial core needle biopsy. These datapoints and findings gave insight into the evolution of this low-grade B-cell lymphoma that ultimately guided treatment.

The patient underwent a left breast lumpectomy for excision of the lymphoma with ultrasound-guided wire localization, where a 4 × 35 × 26 mm specimen was removed.

Preliminary pathology of the specimen shows atypical lymphoid proliferation. Ancillary studies showed neoplastic follicular structures positive for CD20, CD10, and BCL-6. These studies suggest a favorable evolution to DLBCL and follicular lymphoma over marginal zone lymphoma. Following these results, the patient was referred to medical oncology for evaluation for adjuvant chemotherapy and radiotherapy.

## Discussion

Primary breast lymphoma is an incidental diagnosis commonly found during routine breast mass workups in elderly women. This is considered an uncommon finding, representing 0.4-0.5% of all breast cancers [8].

PBL can share many features with incidentally found breast lumps as well as primary and inflammatory breast cancer. Much like the patient we presented, the most common presenting symptom for PBL is a painless breast lump. However, studies show that PBL can also present with clinical symptoms such as nipple discharge, retraction, peau d'orange, and other inflammatory breast cancer symptoms [9].

About 24% of breast lymphomas are clinically asymptomatic and are only identified on screening mammograms, like our presented patient. Unfortunately, this does not confirm a diagnosis, as findings on imaging can mimic breast cancer. Ultrasonographic and mammographic findings of breast lymphoma most commonly show round or oval-shaped intramammary masses, which alone cannot accurately diagnose breast cancer versus breast lymphoma [10]. Since breast cancer and PBL share many clinical features as well as similarities in imaging, the majority of PBL are diagnosed histologically with a biopsy.

In our patient, the initial findings prior to the lumpectomy suggested a low-grade B-cell lymphoma. This is not ideal for the management of the disease. Definitive management of breast lymphoma depends on histological findings, and in this case, they were unclear. Initial pathological findings were found to be positive for HGAL, a gene expressed by tumor cells that mediates cell motility and cytoskeletal regulation [4]. HGAL is most known as a prognostic biomarker for diffuse-large B-cell lymphomas [4,5], as well as Hodgkin's lymphomas [4,6]. It has been found that 50% of breast lymphomas are histologically found to be DLBCLs. These lymphomas are known to commonly have a high proliferation index and a poor prognosis [3,11,12].

Aside from Hodgkin's and DLBCL, breast lymphomas may also be categorized into Burkitt lymphoma, which is more commonly seen in pregnant women and the immunocompromised, as well as the follicular type and mucosa-associated lymphoid tissue (MALT) type marginal zone lymphomas, among other non-Hodgkin's lymphomas [13]. Rarely, breast lymphoma can also present as a T-cell lymphoma [10]. None of which presented in our patient; however, these diseases display the breadth of pathology associated with breast lymphomas. While each type of lymphoma has specific means of management, it is noted that the role of surgery is limited to acquiring tissue for diagnosis. In managing PBL, there is no benefit in mastectomy or other invasive surgical resections [2,3,7].

Since our patient did not have a specific diagnosis with core needle biopsy, along with findings concerning for transformation to DLBCL, a lumpectomy of the mass was the most appropriate next step in management. In the early stages of PBL, a core needle biopsy may not be adequate, and therefore, there is a growing role for surgical excision of tissue in diagnosing breast lymphomas in earlier stages. The standard sequence of management following lumpectomy of PBL is standard anthracycline- and rituximab-based chemotherapy and radiotherapy used in combination [1,7]. The patient will need close follow-up following excision and adjuvant treatment. Most types of PBL are relapsing diseases of the central nervous system [7].

## Conclusions

Having been growing in incidence, representing 0.38-0.70% of all non-Hodgkin lymphomas and 0.4-0.5% of all breast cancers, primary breast lymphomas (PBLs) are a rare but not uncommon finding when screening for breast cancers or masses. These cancers are most commonly diffuse large B-cell lymphomas (DLBCLs), which have a poor prognosis. This rare case shows the management of a low-grade B-cell lymphoma with the potential to differentiate into DLBCL found incidentally on a screening mammogram and appropriate surgical interventions when presented with similar cases. These cancers have been found to respond well to anthracycline and rituximab-based chemotherapy used with or without radiotherapy.
